# The Role of Cognitive Factors in Predicting Balance and Fall Risk in a Neuro-Rehabilitation Setting

**DOI:** 10.1371/journal.pone.0153469

**Published:** 2016-04-26

**Authors:** A. Saverino, D. Waller, K. Rantell, R. Parry, A. Moriarty, E. D. Playford

**Affiliations:** 1 Therapy and Rehabilitation Services, National Hospital for Neurology & Neurosurgery, London, United Kingdom; 2 Institute of Rehabilitation, Fondazione Salvatore Maugeri, Genova, Italy; 3 Wolfson Neurorehabilitation Centre, St Georges Hospital, London, United Kingdom; 4 Research Support Centre, Joint UCL/UCLH/Royal Free Hospital, London, United Kingdom; 5 Institute of Neurology, University College London, London, United Kingdom; Purdue University, UNITED STATES

## Abstract

**Introduction:**

There is a consistent body of evidence supporting the role of cognitive functions, particularly executive function, in the elderly and in neurological conditions which become more frequent with ageing. The aim of our study was to assess the role of different domains of cognitive functions to predict balance and fall risk in a sample of adults with various neurological conditions in a rehabilitation setting.

**Methods:**

This was a prospective, cohort study conducted in a single centre in the UK. 114 participants consecutively admitted to a Neuro-Rehabilitation Unit were prospectively assessed for fall accidents. Baseline assessment included a measure of balance (Berg Balance Scale) and a battery of standard cognitive tests measuring executive function, speed of information processing, verbal and visual memory, visual perception and intellectual function. The outcomes of interest were the risk of becoming a faller, balance and fall rate.

**Results:**

Two tests of executive function were significantly associated with fall risk, the Stroop Colour Word Test (IRR 1.01, 95% CI 1.00–1.03) and the number of errors on part B of the Trail Making Test (IRR 1.23, 95% CI 1.03–1.49). Composite scores of executive function, speed of information processing and visual memory domains resulted in 2 to 3 times increased likelihood of having better balance (OR 2.74 95% CI 1.08 to 6.94, OR 2.72 95% CI 1.16 to 6.36 and OR 2.44 95% CI 1.11 to 5.35 respectively).

**Conclusions:**

Our results show that specific subcomponents of executive functions are able to predict fall risk, while a more global cognitive dysfunction is associated with poorer balance.

## Introduction

In recent years the role of cognitive control on gait, balance and fall risk has received increasing attention[1]. A consistent body of research shows association between cognitive functions and fall risk in the elderly [2] and in neurological conditions traditionally associated with ageing including Alzheimer’s Disease and Parkinson’s Disease [3,4] and more recently in patients with multiple sclerosis [5]. However, limited evidence exists for other neurological conditions such as stroke or traumatic brain injury [6].

All these conditions might share a common denominator of dysregulated cognitive functions affecting control of gait and balance. It has been suggested that gait and balance can no longer be considered simple motor activities but rather complex and goal-oriented activities requiring constant awareness of body movements and the surrounding environment [1]. Executive functions are an umbrella term referring to the cognitive processes relevant to self-monitoring and maintaining purposeful and goal-directed behaviour [7,8] operating in a ‘supervisory’ capacity [9]. Characteristics of executive dysfunction include difficulties with abstract reasoning, planning, decision making, inhibition, perseveration and impulsivity [10].

No single measure of executive function is recognized as a “gold standard” and available tests can only capture specific components of this complex construct [7]. The role of cognitive impairment in fall risk appears well established in the older population [2], where single tests [11,12] and global indices of executive function and attention [13], global cognitive measures [12,14,15] have been associated with increased fall risk. Impairment in executive function and attention has been described in fallers with Parkinson’s Disease [16] and Alzheimer Disease [17,18].

In stroke patients an association between fall risk and cognitive function is unclear with conflicting findings in the literature [19,20], which are based on global measures of cognitive functioning only. However, others [21,22] have found an association between cognition and balance in patients post stroke, using an extensive battery of tests. Liu-Ambrose and colleagues [21] found the Stroop test, measuring cognitive flexibility and response inhibition predicted motor and balance performance. Pahlman and colleagues [22] found that impairment in intelligence and executive function was related to poorer recovery of balance one year after stroke.

D'Orio and colleagues [23] studied neuropsychological predictors of falls in patients with multiple sclerosis, using an extensive battery of tests. Surprisingly they found that a test of verbal recall (California Verbal Learning Test-Second Edition) was the only significant falls predictor. In contrast to D’Orio and colleagues’ findings, other authors showed an association between fall risk and executive functions in individuals with multiple sclerosis [24] [5] measured respectively by the Symbol Digit Modalities Test and by the Trail Making Test. Interestingly the Symbol Digit Modalities Test was associated with fall risk in those with Huntington's disease [25] but not in patients with acquired brain injury [26].

In conclusion there is some evidence describing an association between fall risk and executive function and attention in those with Alzheimer's, Parkinson disease and multiple sclerosis, but the association is not established in other neurological diseases like stroke or traumatic brain injury where such cognitive processes can also be affected. The interpretation of previous results is complicated by the fact that a gold standard measure of executive function and attention does not exist and different neuropsychological tests or indices are not directly comparable.

### Aim of this study

In this study we aim to assess the relationship between different domains of cognitive functions, especially executive functions, and the risk of falling or having impaired balance in adults with long term neurological conditions in an inpatient rehabilitation setting.

Our main hypothesis is that there are components of executive functions associated to the risk of falling in subjects presenting with different neurological conditions.

## Methods

### Study design

A prospective, cohort study conducted in a single centre in the UK.

### Participants

The sample consisted of 114 participants consecutively admitted to the Neuro-Rehabilitation Unit (NRU) at the National Hospital for Neurology and Neurosurgery in London from November 2009 until July 2011. Exclusion criteria were: non-fluency of English, inability to provide informed consent because of severe cognitive or communication difficulties or severe mood or behavioral problems.

Patients presenting with cognitive difficulty or dysphasia were assisted at the stage of consent by the neuropsychologist or speech and language therapist on the NRU. Those unable to consent despite support were not included.

All participants received multidisciplinary assessment and interventions tailored to their needs as part of their usual care. There were no additional interventions as a result of study participation. In the event of a fall, routine practices were put into place to prevent further events.

The study was approved by the East London and the City Research Ethics Committee. Participation was voluntary and written informed consent was obtained.

### Measures and Procedure

#### General measures

The general measures included: age, sex, diagnosis, level of education and years or education, length of admission, level of disability (Functional Independence Measure, FIM, [27] where the total score ranges from 18 to126, with lower scores indicating worse disability) and anxiety/depression (the Hospital Anxiety and Depression Scale [28], HADS, where 2 separate scores are expressed for anxiety and depression ranging from 0 to 21 and where a score of 8 or above represents caseness) on admission. Both the FIM and the HADS are valid and reliable measures commonly used in clinical practise.

#### Outcome variables: fall risk and balance

Falls were recorded prospectively during admission and events were defined as “a sudden and unintentional change in position resulting in an individual landing at a lower level, such as on an object, the floor, or the ground [29].The entire rehabilitation team was asked to complete fall reports if they witnessed a fall or were informed about a fall by a patient. The researchers liaised with the team and the patients weekly to collect fall reports and to complete these with patients if needed. Furthermore the report was completed together with a witness who was present at the moment of the fall to facilitate recall if indicated.

Fall risk was expressed as the binary outcome of being a non- faller or a faller (defined as the participant who experienced at least one fall) and fall rate was calculated as number of falls per 100 days of hospital stay.

Balance was assessed at admission using the Berg Balance Scale [30] (BBS), which is a valid and reliable scale comprising of a set of 14 simple balance related tasks, producing a total score between 0 and 56. Low scores indicate poorer balance. After checking the distribution of the BBS scores and to aid clinical interpretation, the BBS scores were divided into tertiles categories ranging from severe (0–19) to moderate (22–45) and low (46–56) level of impairment. These three categories included an equal number of cases to ensure that the estimates of effect in each group are reasonably precise.

#### Neuropsychological tests

Cognitive variables included an extensive battery of neuropsychological tests assessing different components of executive function, speed of information processing, verbal and visual memory, visual perception, intellectual function and pre-morbid estimate. Some participants were unable to complete some of the tests due to motor, language or visual difficulties. These were coded as missing data.

The following cognitive domains that were assessed and the associated neuropsychological tests are as follows (see Table 1).

**Table 1:** Description of Neuropsychological tests.

Cognitive Domain	Test	Brief Description	Cognitive Processes	Variables
**Executive Function**	**The Stroop Colour Word Test**	Test condition involves reading aloud the colour of the ink the word is printed in rather than reading the words which are also of colours.	Measures selective attention, cognitive flexibility, response inhibition and processing speed.	N correct*,N errors*
	**The Trail Making Test: Part B**	Sequencing and alternating between numbers and letters in order on a page, drawing a line to connect them whilst being timed.	Attention, speed, mental flexibility, visual scanning and visual-motor coordination.	Time(seconds)*, N errors*
	**The Modified Card Sorting Test**	Matching response cards to 4 key cards on the basis of shape, colour or number, using feedback to infer the rule currently in operation.	Abstract reasoning, shift and maintain set, utilize feedback and modulate impulsive responding.	N correct, categories*,N errors*
	**The Controlled Oral Word Association Test**	To generate as many words as possible beginning with specified letters of the alphabet, each within 1 minute whilst adhering to specific rules.	Verbal skills, flexibility of thought including an attention demanding component for self monitoring	N correct*
**Speed of Information Processing**	**The Oral Symbol Digit Modalities Test**	To say aloud numbers that correspond to symbols using a key with a 90 second time limit	Divided attention, visual scanning, perceptual speed and memory.	N correct*
	**The Trail Making Test: Part A**	Sequencing numbers in order on a page drawing a line to connect them whilst being timed.	Attention, speed, visual scanning and visual-motor coordination.	Time(seconds)*
**Verbal Memory**	**Recognition Memory Test for Words**	A 50 item single word study phase followed by a two choice recognition format test phase.	Verbal recognition of printed words	N correct*
	**Doors and People Test: People**	Learning and oral recall of 4 names of people paired with an occupation. 3 learning trials	Verbal learning and recall	Total recall score*
	**Paired Associate Learning Test from the Camden Memory Test Battery**	3 sets of 8 word pairs presented in written format. The participant is asked to name the second word in a pair, when the first one is presented. 2 learning trials.	Cued verbal learning and recall.	N correct for each trial*
**Visual Memory**	**Topographical Recognition Memory Test**	A 30 item single picture study phase followed by a three choice recognition format test phase.	Visual recognition of printed pictures	N correct*
	**Doors and People Test: Shapes**	Learning and recall of 4 shapes, through the reproduction of simple line drawings. 3 learning trials	Visual learning and recall	Total recall score*
**Intellectual Functions**	**Wechsler Adult Intelligence Scale-III**	It consists of a number of verbal and visual subtest	General current intellectual function	Verbal and Performance IQ*
	**National Adult Reading Test-2**	Irregular words reading	Premorbid intellectual functioning	Predicted Full Scale IQ
**Visual perception**	**Incomplete Letters Test (VOSP** ^a^ **subtest)**	The participant has to identify degraded letters	Object perception	N correct
	**Position Discrimination Test (VOSP** ^a^ **subtest)**	The participant has to identify from a choice of 2, which dot is exactly centre in the square	Space perception	N correct

* Variables included in the composite score

^a^ VOSP = Visual Object and Space Perception Battery

For executive function, the Stroop Colour Word Test [31], Part B of the Trail Making Test, The Modified Card Sorting Test [32] and the Controlled Oral Word Association Test [33](FAS Verbal Fluency) were used. Speed of Information Processing was assessed using the Oral Symbol Digit Modalities Test [34] and Part A of the Trail Making Test [35]. For verbal memory the Recognition Memory Test for Words [36], the immediate recall of the People subtest of the Doors and People [37] and the Paired Associate Learning Test from the Camden Memory Test Battery [38] were included. Visual memory was assessed using the Topographical Recognition Memory Test [38], from the Camden Memory Test, and the immediate recall of the Shapes subtest of the Doors and People. Visual perceptual and spatial skills were examined using Incomplete Letter and Position Discrimination subtests of the Visual Object Space Perception Battery [39] (VOSP). Premorbid intellectual functions were estimated using the National Adult Reading Test 2 [40] and current intellectual functions using the Wechsler Adult Intelligence Scale-III [41].

All tests used are valid and reliable measures of cognitive functions, and were administered according to standardised procedures within 10 days of admission across two sessions lasting 1–1.5 hours each on average. The administration order of tests was the same for all participants but extra sessions were arranged, if additional time was required. The first session was administered by one of two clinical psychologists with expertise in neuropsychology, while the second was administered by two researchers, specifically trained by the psychologists. Responses were scored according to standardised procedures.

Visual perceptual and spatial tests and estimated pre-morbid intelligence were used to describe baseline characteristics between fallers and non-fallers. All other tests were used to predict the outcomes of interest (fall and balance). Tests assessing the same cognitive domain were combined together to form a composite score.

### Statistical methods

#### Derivation of composite scores

We used the Z-score to transform all variables prior to combining them into a single composite score. The Z-score is the number of standard deviation units a person's score is below or above the average score, provided the underlying measurements are continuous or count variables. The directions of Z-scores for different test variables were adjusted so that in all cases higher Z-scores corresponded to better function. Composite score was derived based on the average of the Z-transformed scores, if at least 50% of the tests involved were not missing. Missing composite scores were replaced by the group (faller and non-faller) average scores. We carried out additional sensitivity analyses based on non-missing cases and using the minimum and maximum group number to replace missing data. The unit-weighted composite scores were then used as predictor variables in subsequent analysis. Details of the tests used to derive the composite scores are given in Table 1.

#### Analysis

Numerical data were summarised using mean and standard deviation or median and range, depending on data distribution. Categorical data were summarised using count and percentages.

We used a modified Poisson regression model with robust error variance to assess the relationship between cognitive functions and risk of falls, offset to length of stay in hospital. The use of robust estimation deals with the problem of overestimation when the Poisson regression is applied to binary data. This method also provides estimates in terms of relative risk which are easier to interpret compared to odds ratio and has also been shown to be reliable even with a small sample size [42]. We performed an unadjusted analysis and adjusted analysis incorporating potential confounders (age, sex and number of education years) for each of the outcomes as suggested by previous literature [21], [23].

We fitted ordinal regression based on cumulative odds model to assess the relationship between the BBS categories and cognitive functions, offset to length of stay in hospital. This technique generates a single odds ratio for ordered categorical outcomes and assumes that the odds ratios are not dependent on the particular cut-points chosen [43]. Odds ratio from this model estimates the probability of having a particular level of balance impairment (mild) versus moderate or severe. We checked the proportional assumption required for the model to be valid using Brant test, which is available in Stata [44].

In order to assess the strength of association, estimates from models are reported along with their 95% confidence interval. Analysis was carried out in Stata V12 [45]. No adjustment for multiple testing was carried out. Therefore significant findings need to be interpreted with caution.

## Results

### Population characteristics

The flow of patients into the study and the characteristics of those agreeing and declining study participation are described elsewhere [46].

General characteristics of the studied population are reported in Table 2.

**Table 2:** Description of patient characteristics by fall status (n = 114).

	Variable	Fallers (n = 34) ^a^ mean ± SD or count (%)	Non-fallers (n = 80) mean ± SD or count (%)	All subjects (n = 114) mean ± SD or count (%)
Person days*		1769	3584	5353
**Demographic**	Age (years)	43.5 ± 13.0	45.0 ± 17.3	44.5 ± 16.2
	Female	13 (38.2)	37(46.2)	50 (44%)
	**Education qualification**			
	None	1 (3)	6 (8)	7 (6)
	GCSE	8 (23)	18 (22)	26 (23)
	A level	6 (18)	13 (16)	19 (17)
	Undergraduate	5 (15)	18 (22)	23 (20)
	Postgraduate	1 (3)	7 (9)	8 (7)
	Missing	13 (38)	18 (22)	31 (27)
	**Years of educations**	14 (2)	14 (3)	14 (3)
**Previous history**	**Diagnosis**			
	Multiple sclerosis	9 (26)	23 (29)	32 (28)
	Stroke	9 (26)	16 (20)	25 (22)
	Spinal cord injury	4 (12)	13 (16)	17 (15)
	Others**	12 (35)	28 (35)	40 (35)
	**Diagnosis compatible with cognitive impairment**			
	yes	25 (73)	57 (71)	82 (72)
	no	9 (27)	23 (29)	32 (28)
**Hospital admission**	**Length of stay** ^b^ (days)	52.0 ± 25.7 ^b1^	44.8 ± 27.4 ^b2^	46.8 ± 27.3 ^b3^
**Functional level**	**FIM** ^c^ **total**	91.5 ± 16.3 ^c1^	94.3 ± 19.2 ^c2^	93.5 ± 18.4 ^c3^
	**Visuo-perceptual function**			
	VOSP ^g^ incomplete letters (normal)	32 (99)	66 (83)	98 (99)
	VOSP ^g^ position discrimination (normal)	30 (88)	67(84)	97 (98)
	**Intellectual functioning**			
	Premorbid (NART)^d^	111.3 ± 10^d1^	105.1 ± 12.4^d2^	107.1 ± 11.9 ^d3^
**Anxiety and depression**	**HADs**			
	anxiety ^e^	6.4 ± 3.5^e1^	5.7 ± 4.1 ^e2^	6 ± 3.9 ^e3^
	depression ^f^	5.6 ± 3.6 ^f1^	5.4 ± 3.8 ^f2^	5.5 ± 3.7 ^f3^

^*a*^16 (47%) were recurrent fallers (who had more than one fall); 18 (52%) were single fallers

^b^ LOS = length of stay median (range): ^b1^ 46.5 (17–110), ^b2^ 38.5 (6–134), ^b3^ 39.5 (6–134)

^c^ FIM = functional Independence Measure median (range): ^c1^ 94 (59–120), ^c2^ 98 (48–123), ^c3^ 97 (48 to 123)

^d^ NART = National Adult Reading Test median (range): ^d1^ 114 (89–124), ^d2^ 105 (81–128), ^d3^ 110 (81–128)

^e^ HAD = Hospital Anxiety and Depression Scale- anxiety median (range): ^e1^ 6 (1–13), ^e2^ 5 (0–16), ^e3^ 5 (0–16)

^f^ HAD = Hospital Anxiety and Depression Scale- depression median (range):^, f1^ 5 (1–15), ^f2^ 5 (0–14), ^f3^ 5(0–15)

^g^ VOSP = Visual Object and Space Perception Battery. % out of 99

* Fall rate per 100 days of patient hospital stay = 71 (number of falls)/5353(total person days) x 100 = 1.33

** Others: miscellaneous peripheral and central nervous system illnesses.

Overall, the mean (SD) age was 44.5 (16.2) years and less than half were female (n = 50). The majority of participants had some education with a mean (SD) number of years of education of 14 (3) years. Over a quarter of participants were educated to at least undergraduate level (n = 31). The three most common diagnoses, which account for 65% (74/114) of all diagnoses include: multiple sclerosis 28% (32), stroke 22% (25) and spinal cord injury 15% (17). Overall 72% (82) of all diagnoses were classified as compatible with a certain degree of cognitive impairment, being the anatomical lesion in the central nervous system above the spinal cord level.

The total FIM score ranged from 48 to 123, with a mean (SD) of 93.5 (18.4). Visual perceptual and spatial functions were normal in almost all participants (99% and 98% respectively). The mean (SD) estimated premorbid intellectual functioning based on the NART IQ was 107 (12). The mean (SD) depression and anxiety scores were 6 (3.9) and 5.5 (3.7) respectively.

The mean (SD) of the length of stay was 46.8 (27.3) days, ranging from 6 to 134 days. The fallers group tended to be younger, male, with a longer hospital stay, a lower FIM score and a higher NART. Although, there were no statistically significant differences between fallers and non- fallers with respect to age, sex, education, diagnosis, length of stay, level of disability, visual perceptual/spatial function and pre morbid intelligence estimate (Table 2).

### Falls events

Almost a third of the patients (34/114) experienced a fall with a total fall count of 71. Fall rate (95% CI) per 100 days hospital stay was 1.33 (1.04 to 1.67) (Table 2). Falls characteristics are described in an earlier published paper [46].

### Cognitive functions and fall risk

Table 3 reports medians and ranges of the neuropsychological test raw scores by fallers and non- fallers, showing a lower but not statistically significant performance in verbal (recognition and recall) and visual (recall) memory tests in fallers.

**Table 3:** Neuropsychological tests raw scores by fallers and non fallers.

	Faller Median* (range)	Non-faller Median* (range)	Medians* difference [95% CI] **
**Executive function**			
The Stroop Colour Word Test	88 (25–112)	86 (7–112)	-6 [-18 to 4]
The Stroop Colour Word Test errors	0 (0–16)	0 (0–21)	0 [0 to 0]
The Trail Making Test: Part B	120 (40–417)	117 (38–566)	-6 [-40 to 26]
The Trail Making Test: Part B-errors	0 (0–6)	0 (0–7)	0 [0 to 0]
The Modified Card Sorting Test	6 (1–6)	6 (1–6)	0 [0 to 0]
The Modified Card Sorting Test-errors	7 (0–25)	6 (0–29)	0 [-2 to 2]
The Controlled Oral Word Association Test	35 (11–59)	32 (7–87)	4 [-4 to 10]
**Speed of processing**			
The Oral Symbol Digit Modalities Test	41 (11–66)	38 (2–79)	-2 [-8 to 4]
The Trail Making Test: Part A	47 (18–224)	52 (18–221)	0 [-12 to 10]
**Verbal memory**			
Recognition Memory Test for Words	47 (34–50)	48 (26–50)	0 [0 to 2]
Doors and People Test: People	25 (8–36)	28 (3–36)	2 [-2 to 4]
Paired Associate Learning Test: part 1	21 (9–24)	22 (6–24)	0 [0 to 2]
Paired Associate Learning Test: part 2	24 (18–24)	24 (7–24)	0 [0 to 0]
**Visual memory**			
Topographical Recognition Memory Test	27 (11–30)	26 (13–30)	0 [-2 to 2]
Doors and People Test: Shapes	33 (10–36)	34 (8–36)	0 [0 to 4]
**General intelligence**			
WAIS IQ verbal	102 (61–130)	98 (73–140)	-2 [-8 to 8]
WAIS IQ performance	92 (65–128)	90 (58–140)	-2 [-10 to 8]

* Medians are reported instead of means because data distribution is skewed

** P-values based on Mann-Whitney test were > 5%.

Confidence intervals represent the generalized Hodges-Lehmann median difference

Table 4 shows the results of the regression model where raw scores of the neuropsychological tests as well as the composite scores of the different cognitive domains are used to predict fall and balance (BBS categories).

**Table 4:** Relationship between cognitive factors and risk of fall and Berg Balance Scale.

Factors	Fall risk ^4^		Berg Balance Scale categories ^5^	
Cognitive domains and tests	Unadjusted	Adjusted ^1^	Unadjusted	Adjusted ^1^
	Risk (95% CI)	Risk (95% CI)	OR (95% CI)	OR (95% CI)
**Executive function- composite** ^2^	0.96 [0.65–1.40]	1.024 [0.58–1.80]	2.42 [1.19–4.94]	2.74 [1.08–6.94]*
The Stroop Colour Word Test ^3^	1.00 [0.99–1.02]	1.01 [1.00–1.03]*	1.00 [0.98–1.02]	1.00 [0.98–1.03]
The Stroop Colour Word Test errors ^3^	0.95 [0.88–1.04]	0.87 [0.75–1.02]	0.91 [0.80–1.03]	0.90 [0.79–1.03]
The Trail Making Test: Part B ^3^	0.99 [0.99–1.00]	1.00 [0.99–1 00]	0.99 [0.99–1.00]	0.99 [0.98–0.99]*
The Trail Making Test: Part B-errors ^3^	1.07 [0.88–1.30]	1.23 [1.03–1.49]*	0.79 [0.57–1.10]	0.65 [0.37–1.14]
The Modified Card Sorting Test ^3^	1.00 [0.85–1.18]	1.04 [0.78–1.39]	1.13 [0.81–1.57]	1.06 [0.65–1.70]
The Modified Card Sorting Test-errors ^3^	0.99 [0.95–1.04]	1.00 [0.95–1.05]	0.97 [0.90–1.04]	0.97 [0.89–1.06]
The Controlled Oral Word Association Test ^3^	0.99[0.96–1.01]	0.99 [0.96–1.02]	1.03 [0.99–1.07]	1.03 [0.98–1.09]
**Speed of information processing- composite** ^2^	1.07 [0.74–1.54]	1.04 [0.65–1.65]	1.99 [1.10–3.58]*	2.72 [1.16–6.36]*
The Oral Symbol Digit Modalities Test ^3^	1.00 [0.98–1.03]	1.01 [0.98–1.04]	1.04 [1.01–1.08]*	1.07 [1.03–1.23]*
The Trail Making Test: Part A ^3^	0.99 [0.99–1.00]	0.99 [0.99–1.00]	0.98 [0.97–0.99]*	0.98 [0.96–1.00]**
**Verbal Memory-composite** ^2^	1.04 [0.78–1.38]	1.06 [0.64–1.73]	1.80 [1.06–3.04]*	1.87 [0.90–3.89]
Recognition Memory Test for Words ^3^	1.01 [0.95–1.07]	1.01 [0.90–1.14]	1.11 [1.01–1.23]*	1.16 [0.98–1.37]
Doors and People Test: People ^3^	1.00[0.96–1.03]	1.01 [0.95–1.07]	1.07 [1.01–1.14]*	1.08 [0.99–1.18]**
Paired Associate Learning Test: part 1 ^3^	1.00[0.94–1.05]	0.99 [0.91–1.08]	1.07 [0.96–1.19]	1.07 [0.94–1.22]
Paired Associate Learning Test: part 2 ^3^	1.04 [0.96–1.12]	1.05 [0.94–1.18]	1.15 [0.99–1.34]	1.12 [0.92–1.34]
**Visual Memory- composite** ^2^	0.92 [0.66–1.26]	0.92 [0.61–1.4]	2.00 [1.14–3.49]*	2.44 [1.11–5.35]*
Topographical Recognition Memory Test ^3^	0.99 [0.93–1.05]	1.00[0.91–1.09]	1.13 [1.01–1.27]*	1.19 [1.02–1.39]*
Doors and People Test: Shapes ^3^	0.99 [0.95–1.02]	0.98 [0.94–1.03]	1.08 [1.01–1.17]*	1.09 [0.98–1.2]
**Intelligence composite** ^2^	1.15 [0.77–1.73]	1.16 [0.64–2.10]	0.82 [0.42–1.58]	0.87 [0.36–2.12]
WAIS IQ verbal ^3^	0.99 [0.98–1.00]	0.99 [0.97–1.02]	0.99 [0.96–1.02]	0.95 [0.90–1.00]
WAIS IQ performance^3^	1.00 [0.98–1.02]	0.99 [0.97–1.02]	0.99 [0.96–1.03]	1.02 [0.97–1.07]

* test statistically significant (the confidence interval does not crosses 1 and p<0.05);

** test with borderline significance (p value = 0.05).

^1^ Adjusted by age, sex and years of education.

^2^ composite z-scores

^3^ raw scores

^4^ fall rates set off by length of stay

^5^ severe (0–19) moderate (22–45), and mild (46–56) balance impairment.

None of the composite scores predicted of fall. However two of the executive function scores were significantly associated with fall risk, the Stroop Colour Word Test (IRR 1.01, 95% CI 1.00–1.03) and the number of errors on part B of the Trail Making Test (IRR 1.23, 95% CI 1.03–1.49), after adjusting for sex, age and years of education. The fewer the number of correct items on the Stroop Colour Word Test and the more errors made on part B of the Trail Making Test, meant there was a greater likelihood of becoming a faller.

The distribution of missing data showed that the proportion of missing data was higher in the non-faller group compared to the faller group in all cognitive domains but the trend was consistent between the two groups. The proportion of missing was highest in the intelligence domain followed by speed, visual memory, verbal memory and executive function. No pattern could be discerned through additional sensitivity analyses based on complete cases and using the minimum and maximum group number to replace missing data.

### Cognitive functions and balance

Results for the adjusted analysis showed that all composite scores with the exception of verbal memory and intelligence were significant associated with the BBS (Table 4). A better performance in the executive function, speed of information processing and visual memory domains (higher composite scores) resulted in 2–3 times increased likelihood of having better balance (OR 2.74 CI 95% 1.08 to 6.94, OR 2.72 CI 95% and OR 2.44 CI 95% 1.11 to 5.35 respectively).

The raw scores of three tests were significant predictors of balance after adjustment for age sex and education. A longer time (worse performance) to complete part A and B of the Trail Making Test (executive function and speed of information processing respectively), was significantly associated with worse balance (OR 0.98 95% CI 0.97–0.99 and OR 0.99 95% CI 0.98–0.99 respectively). On the other hand a bigger number of correct responses on the Oral Symbol Digit Modalities Test (speed of information processing) and the Topographical Recognition Memory Test (visual memory) were significantly associated with better balance (OR 1.07 95% CI 1.03–1.23 andOR 1.19 95% CI 1.02–1.39, respectively).

## Discussion

Overall 30% of the patients admitted to our unit fell at least once during their stay and fall rate was 1.33 per 100 days of patient hospital stay. Our study shows that two tests of executive function: the Stroop Colour Word Test and the number of errors on the part B of the Trail Making Test, were significant predictors of risk of fall in our population. Additionally, we identified different measures of executive function, speed of information processing and memory able to significantly predict poor balance performance.

The incidence of falls in our study appears substantially higher in comparison to the rate of 7.9/1000 described in an older adults hospital stay [47] and the rate of 6.7 /1000 described in a general inpatient rehabilitation ward [48]. Our higher rate might be due to the design of our study, possibly less biased by recall errors, or to differences in participant and environment characteristics.

Consistent with previous research we found an association between specific cognitive functions and fall risk and balance. An explorative analysis comparing the raw scores of neuropsychological tests between fallers and non-fallers suggested a tendency in fallers to have increased memory difficulties.

The fact that only the Trail Making Test B number of errors and the Stroop Colour Word Test, amongst all the other measures of cognitive functions, were able to predict fall risk, suggests that difficulties in response inhibition, attention and switching under timed conditions may have been the key components of executive functions impacting on fall risk in our population. A possible interpretation is that people with difficulties in response inhibition and switching may have difficulties dealing with distracters or competing responses when walking or moving around by wheelchair or transferring from different positions, resulting in a fall. Consistent with our results, the role of executive function in predicting fall risk has been previously described in an elderly population [2,13,49] as well as in patients with Parkinson’s Disease [16], Alzheimer Disease [17,18] and multiple sclerosis [5]. In particular, previous studies have shown that individuals at risk of falls do worse on the Trail Making Test B [5][11][50][51][52]. However, these previous studies found a correlation with the time to complete the test or used the difference between Trail Making Test Part B and Trail Making Test Part A as the outcome of interest [5][51], while no data is available on the number of errors made.

Interestingly D’Orio and colleagues [23] studied the risk of falls in adults with multiple sclerosis using an extensive battery of neuropsychological tests but did not find any test of executive function able to predict falls. Unfortunately this author did not use the Stroop Test or the Trail Making Test, thus not allowing comparison with our results.

The association between the Symbol Digit Modalities and falls was previously described in patients with Huntington’s disease [25] and patients with multiple sclerosis [24], but was not replicated in our study. This may be due to differences in population, setting and methodology used by the other authors.

In our population the executive function composite score was associated with a 2–3 times increased chance of having better balance and part B of the Trail Making Test was also significantly associated with balance performance. Previous studies on post stroke patients [21,22] have described the same association between executive function and balance, which can be explained by the theory of executive control over balance and gait [1]. All the measures of speed of information processing included in our study were significantly associated with balance and the result obtained with the Symbol Digit Modalities Test is commensurate with previous studies based on patients with multiple sclerosis and Huntington’s disease [24,25]. It is possible that participants with impaired speed of information processing are unable to select an appropriate motor response to react [24]or to anticipate a balance perturbation. Surprisingly we also found an association between balance and memory. Although a previous study [23] described poor verbal memory as a significant predictor of falls, the role of memory on balance and fall risk has no clear scientific explanation and may deserve further research.

We suggest, as an alternative hypothesis, that our findings might simply reflect an underlying severe neurological deterioration affecting both global cognition (including memory, speed of information processing and executive functions) and balance, with no specific effect on balance by the different cognitive processes.

In summary, our results show that specific subcomponents of executive function are able to predict fall risk, while a more global cognitive dysfunction is associated with poorer balance.

We suggest two possible interpretations to explain the different results obtained for fall risk and balance. The first being that people with poor balance and cognitive difficulties, can still prevent falls avoiding dangerous actions, unless they have a specific cognitive problem affecting switching ability or inhibition of more automatic responses. Therefore only the participants showing uninhibited responses may have incurred falls. The second is that patients with poor balance and globally impaired cognitive functions might have been more easily recognized as potential fallers and have received routine interventions by the team to prevent falls (i.e. more supervision). Moreover falls of participants with uninhibited behaviors are difficult to prevent unless receiving constant supervision.

Our study presents different limitations. A selection bias might have occurred due to a higher proportion of females declining participation in the study and a higher proportion of males taking part in the study. However there is no evidence in the literature of an effect of gender on fall risk.

The decision to adopt broad inclusion criteria inevitably brought missing data due to the presence of participants who were unable to perform certain tests due to motor, visual or speech difficulties. However, missing data was not due to a cognitive deficit in the domain being assessed and most statistical models, except the one relating to the Intelligence domain, had sufficient observations to support the number of variables considered in the model. Although the proportion of missing data was higher in the faller group, additional sensitivity analyses to impute missing data showed an overall consistency of results.

The predictive power of the identified tests is small, suggesting only a partial role of cognitive functions in determining balance problems and fall risk, which was expected considering the well-known multifactorial origin of falls [52].

Finally we might have underestimated the true effect of cognitive difficulties by including in our study patients not expected to have cognitive problems and potentially with a different risk profile such as those with spinal cord conditions.

The main strengths of our study are that our overall sample size was relatively large compared to previous similar studies and looked at a more diverse neurological population. The focus of our research is on the functioning of the subjects and not on the aetiology of the neurological diagnosis; executive functioning is considered the key common denominator across different conditions. Small numbers by different diagnosis in our population do not allow drawing conclusions by diagnosis.

In addition, we used an extended battery of neuropsychological tests and prospective collection of falls.

Our study may have significant implications in managing fall risk in young adults with neurological conditions, describing two simple cognitive tests which could be used in addition to other instruments of multifactorial risk assessment to identify potential fallers and to target interventions for those at risk. However our results have to be interpreted with caution due to the limitations described and further research is needed to confirm the role of specific subcomponents of executive functions and specific tests (the Stroop Test and the Trail Making Test in particular) in determining fall risk.

Longitudinal studies following up the patients in the community could add further understanding regarding the role of environment and setting.

## Conclusion

Our study is the first study to highlight the importance of specific components of executive function on fall risk in a population of young adults with neurological conditions during their stay in a neuro-rehabilitation unit.
